# Differential protection by wildtype vs. organelle-specific Bcl-2 suggests a combined requirement of both the ER and mitochondria in ceramide-mediated caspase-independent programmed cell death

**DOI:** 10.1186/1748-717X-4-41

**Published:** 2009-10-09

**Authors:** Andrea Deerberg, Justyna Sosna, Lutz Thon, Claus Belka, Dieter Adam

**Affiliations:** 1 Institut für Immunologie, Christian-Albrechts-Universität Kiel, 24105 Kiel, Germany; 2 Klinik und Poliklinik für Strahlentherapie und Radioonkologie, Ludwig-Maximilians Universität München, 81377 München, Germany

## Abstract

**Background:**

Programmed cell death (PCD) is essential for development and homeostasis of multicellular organisms and can occur by caspase-dependent apoptosis or alternatively, by caspase-independent PCD (ciPCD). Bcl-2, a central regulator of apoptosis, localizes to both mitochondria and the endoplasmic reticulum (ER). Whereas a function of mitochondrial and ER-specific Bcl-2 in apoptosis has been established in multiple studies, corresponding data for ciPCD do not exist.

**Methods:**

We utilized Bcl-2 constructs specifically localizing to mitochondria (Bcl-2 ActA), the ER (Bcl-2 cb5), both (Bcl-2 WT) or the cytosol/nucleus (Bcl-2 ΔTM) and determined their protective effect on ceramide-mediated ciPCD in transiently and stably transfected Jurkat cells. Expression of the constructs was verified by immunoblots. Ceramide-mediated ciPCD was induced by treatment with human recombinant tumor necrosis factor and determined by flow cytometric measurement of propidium iodide uptake as well as by optical analysis of cell morphology.

**Results:**

Only wildtype Bcl-2 had the ability to efficiently protect from ceramide-mediated ciPCD, whereas expression of Bcl-2 solely at mitochondria, the ER, or the cytosol/nucleus did not prevent ceramide-mediated ciPCD.

**Conclusion:**

Our data suggest a combined requirement for both mitochondria and the ER in the induction and the signaling pathways of ciPCD mediated by ceramide.

## Background

The survival and homeostasis of multicellular organisms critically depends on programmed cell death (PCD) to correctly remove cells that are potentially harmful or which have fulfilled their function. Although caspase-dependent apoptosis, the most well-studied form of PCD, represents the principal suicide program in most physiological settings [1], many cells have the ability to commit suicide by caspase-independent modes of PCD (ciPCD) [2]. ciPCD fulfills vital functions in processes such as the negative selection of lymphocytes, the termination of immune responses, embryonic removal of interdigital webs, regulation of bone growth, ovulation, and cellular turnover in the intestine [3]. Furthermore, ciPCD has been implicated the pathology of hyperacute shock [4], pancreatitis [5,6], cerebral and myocardial ischemia-reperfusion injury, epilepsy, Alzheimer's disease and other inflammatory injuries, as well as in the destruction of cells by pathogens such as HIV, vaccinia virus, Shigella and Salmonella [3,7]. In contrast to apoptosis, the molecular mechanisms of ciPCD are just beginning to be unraveled, e. g. by the recent identification of RIP3, enzymes of the energy metabolism, Nox1, CYLD, Bmf, and cathepsin and calpain proteases as candidate mediators of ciPCD [2,8-10]. However, and despite these advances, a coherent picture of the molecular steps in ciPCD is still lacking.

Mitochondria have been identified not only as essential elicitors of apoptosis, but also of ciPCD, e.g. by release of proteins such as AIF, EndoG and HtrA2/OMI, as well as by production of reactive oxygen species (ROS) [2,11-13]. It has been proposed that excess formation of ROS triggers ciPCD by activation of the DNA repair enzyme PARP, followed by intracellular depletion of NAD^+ ^and ATP, nuclear translocation of AIF and finally, death [14]. Similar to mitochondria, the endoplasmic reticulum (ER) - as the main site for critical cellular functions such as protein folding, lipid biosynthesis, and calcium storage in the cell - has also been implicated in the induction of both apoptosis [15] and ciPCD [2,12,13]. In fact, the ER may play a key role in certain types of ciPCD, as intracellular calcium influx caused by ER stress induces activation of calpains, a family of calcium-dependent cytosolic proteases that can elicit ciPCD [2].

Members of the Bcl-2 family of proteins have long been recognized as central regulators of mitochondrial apoptosis, primarily by controlling the permeabilization of the outer mitochondrial membrane [15]. They have also been found localized at the ER, where they regulate apoptosis in response to a range of cellular stresses, and also in the nucleus [15], where they may fulfill yet unknown functions. Similar to the established protective functions of mitochondrial Bcl-2, expression of Bcl-2 at the ER has been shown to protect cells from apoptosis, e. g. by regulating the release of proapoptotic calcium, reducing calcium-uptake by mitochondria and subsequent calpain-dependent apoptosis [15]. The role of Bcl-2 in mitochondrial vs. ER-mediated apoptosis has been further addressed in a number of studies by the use of Bcl-2 constructs that specifically localize to mitochondria or to the ER [16-23]. In these studies, Bcl-2 localizing to the ER was shown to interfere with apoptosis induction by some (c-myc overexpression, etoposide, staurosporine, tunicamycin, brefeldin A, ceramide, ionizing radiation, thapsigargin, Bax, Bad) but not all stimuli (reviewed in [24,25]).

In contrast to this relative wealth of available data on apoptosis, the role of ER- vs. mitochondrially localized Bcl-2 in ciPCD has not been investigated so far. Here, we utilize ciPCD elicited by ceramide as a model system, a lipid second messenger that has been recognized as important in radiation-induced elimination of tumor cells [26,27]. We demonstrate for the first time that in contrast to wildtype Bcl-2, restricted expression of Bcl-2 solely at mitochondria-, the ER-, or the cytosol/nucleus is insufficient to prevent ciPCD. Therefore, our data suggest a combined requirement of mitochondria and the ER in ceramide-mediated ciPCD and implicate the existence of a molecular crosstalk between both organelles.

## Methods

### Reagents and constructs

Highly purified human recombinant TNF (hTNF) was supplied by BASF Bioresearch. Benzyloxycarbonyl-Val-Ala-Asp-fluoromethylketone (zVAD-fmk) was obtained from Bachem. Cycloheximide (CHX) was purchased from Sigma. Expression constructs cloned in the vector pRc/CMV (Invitrogen) encoding wildtype Bcl-2 (Bcl-2 WT), cytosolic Bcl-2 lacking the transmembrane domain (Bcl-2 ΔTM) and Bcl-2 mutants restricted to the outer mitochondrial membrane (Bcl-2 ActA) or to the endoplasmic reticulum (Bcl-2 cb5) were kindly provided by B. Leber (McMaster University, Hamilton, Canada).

### Cell culture and transfections

Wildtype human leukemic Jurkat cells were originally obtained from the American Type Culture Collection. Jurkat cells stably transfected with the plasmid pSFFV-Bcl-2, overexpressing full length human Bcl-2 at levels 10-20-fold over untransfected cells [28] were kindly provided by S. Korsmeyer (Harvard Medical School, Boston, USA). Cells were maintained in a mixture of Click's/RPMI 1640 (50/50% v/v) supplemented with 10% v/v FCS, 2 mM glutamine and 50 μg/ml each of streptomycin and penicillin in a humidified incubator containing 5% w/v CO_2_. Transient transfections of wildtype Jurkat cells were performed by Amaxa nucleofection (Lonza Cologne), using solution V and program C-16. Jurkat cells stably expressing the various versions of Bcl-2 in pRc/CMV were obtained by electroporation using a Gene pulser II (Bio-Rad) followed by selection with Geneticin (Invitrogen) and were used as pool transfectants.

### Caspase assays

Cells were lysed in a buffer containing 10 mM Hepes pH 7.4, 142 mM KCl, 5 mM MgCl_2_, 1 mM EGTA, 0.2% v/v NP40, 1 mM DTT and 2 mM Pefabloc. To generate positive controls for activation, cytosolic extracts of untreated cells were equilibrated for 1 h at 30°C after the addition of 1 mM dATP and 10 μM cytochrome c to permit activation of caspases and subsequent cleavage of substrate proteins. To measure caspase activity, 100 μl of caspase buffer (20 mM Pipes, 100 mM NaCl, 10 mM DTT, 1 mM EDTA, 0.1% w/v CHAPS, 10% w/v sucrose, pH 7.2) containing 100 μM zDEVD-afc or zIETD-afc (Calbiochem) were added to 5 μl of cytosolic extract (50 μg protein) and incubated at 37°C. The release of afc was measured as emission at 505 nm upon excitation at 405 nm using a Labsystems Fluoroskan II fluorimeter equipped with a thermostated plate reader.

### Microscopy

For documentation of cell morphology, images from unfixed cells were obtained using an Axiovert 100 microscope (Zeiss) and a DS-5 M camera (Nikon).

### Cytotoxicity assays

**Figure 1:**
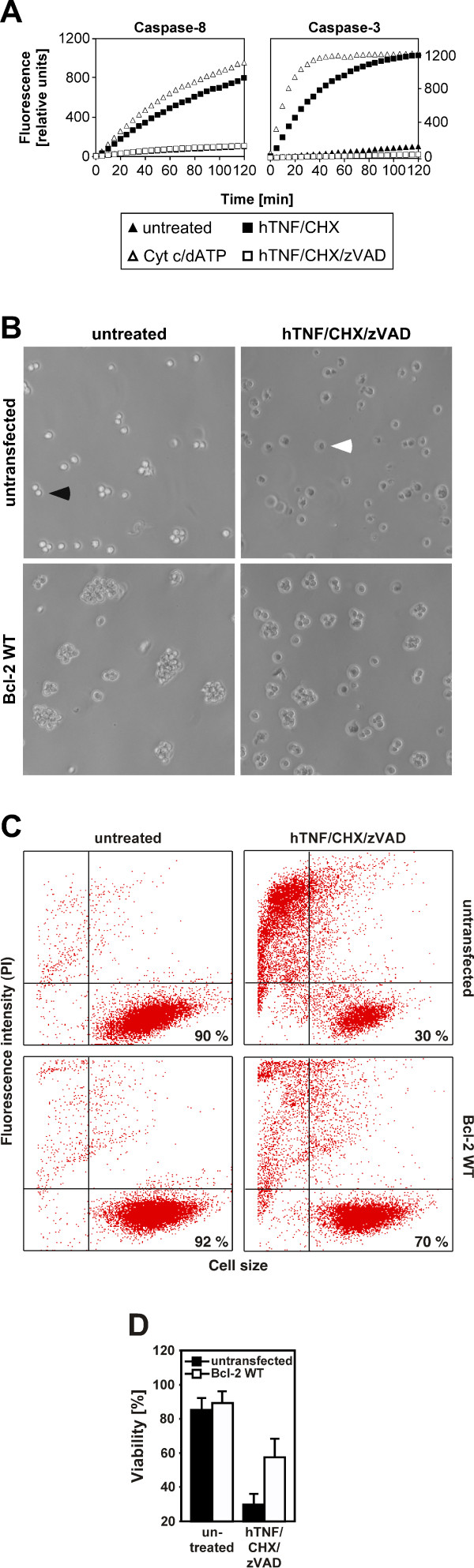
**Wildtype Bcl-2 protects from ceramide-induced ciPCD**. (A) Activity of caspase-8 and -3 in wildtype Jurkat cells in response to TNF/CHX/zVAD inducing ciPCD or TNF/CHX as a proapoptotic stimulus. Cells were incubated with 100 ng/ml hTNF in combination with 2 μg/ml CHX and/or 50 μM zVAD-fmk for 4 h before activation of caspases -8 and -3 was determined by measuring the cleavage of fluorogenic substrates (zIETD-afc and zDEVD-afc) over 120 minutes. Prior to stimulation, cells were preincubated with 50 μM zVAD-fmk for 30 min (for stimulations with TNF/CHX/zVAD) or medium (stimulations with TNF/CHX). For positive control, caspases were activated in vitro by adding cytochrome c and dATP (Cyt c/dATP) to the cell extracts. (B) Wildtype (untransfected) Jurkat cells and Jurkat cells overexpressing pSFFV-Bcl-2 (Bcl-2 WT) were left untreated or stimulated with 100 ng/ml hTNF in combination with 5 μg/ml CHX and 50 μM zVAD-fmk for 20 h before micrographs of the cells were taken to document their morphology. Prior to stimulation, the cells were preincubated for 60 min with 50 μM zVAD-fmk. As representative examples, one healthy cell and one cell undergoing ciPCD with necrosis-like morphology are marked by a black or a white arrow, respectively. (C) In parallel, uptake of PI was determined by flow cytometry as a marker for loss of plasma membrane integrity (see „Materials and Methods‟). The percentage of viable cells (PI-negative, large) is indicated in the lower right quadrants of the dot plots. One representative experiment out of three performed is shown. (D) Quantification of cell viability data. The bar graphs represent the means from all three independent experiments, error bars indicate the respective standard deviations.

For flow cytometric measurement of cell death, cells were seeded in six-well plates at 5 × 10^5 ^cells/well. Following treatment, cells were collected by centrifugation and resuspended in PBS/5 mM EDTA containing 2 μg/ml propidium iodide (PI), and the red fluorescence was measured on a FACSCalibur flow cytometer (BD Biosciences). Since we observed that measurement exclusively of PI-positive cells did not account for a significant fraction of highly disintegrated dead cells that - due to diffusion of the dye - had already become PI-negative again (shown e. g. in Fig. 1C, right panels, lower left quadrants), we alternatively measured the fraction of large, PI negative cells (lower right quadrants) which represents viable, intact cells. For each measurement, a total of 10.000 cells was analyzed.

### Immunoblots

Cells were collected and lysed in TNE buffer (50 mM Tris pH 8.0, 150 mM NaCl, 1% v/v NP40, 2 mM EDTA) containing 10 μg/ml pepstatin/aprotinin/leupeptin, 1 mM sodium orthovanadate and 5 mM NaF. After removal of insoluble material by centrifugation at 10.000 × g and 4°C for 15 min, the protein concentration of the supernatants was measured using a BCA assay (Pierce). 30 μg of cell protein per lane were resolved by electrophoresis on 12.5% w/v SDS polyacrylamide gels (SDS-PAGE). After electrophoretic transfer to Protran nitrocellulose (Whatman), reactive proteins were detected using a monoclonal IgG1 antibody specific for human Bcl-2 (sc-7382, Santa Cruz) and the ECL detection kit (GE Healthcare).

## Results

### Wildtype Bcl-2 protects from ceramide-mediated ciPCD

In a first set of experiments, we examined the ability of full-length, wildtype Bcl-2 to protect human leukemic Jurkat T cells from ceramide-mediated ciPCD elicited by TNF-R1, a system that we have intensively characterized in previous studies. In this system, ceramide is generated exclusively by the lipase acid sphingomyelinase, and clonogenicity of tumor cells is dramatically reduced by activation of this pathway [29-32]. To evaluate the role of wildtype Bcl-2, we treated wildtype Jurkat cells with TNF in combination with the broad-spectrum caspase-inhibitor zVAD and the protein biosynthesis inhibitor CHX (to sensitize the cells) and compared their survival to a Jurkat cell line that stably overexpresses wildtype Bcl-2 in the vector pSFFV-neo [28]. We have previously confirmed that this treatment induces PCD exclusively by caspase-independent mechanisms, but not by apoptosis [31,32]. In this study, we additionally verified this by measuring the activities of the initiator-caspase 8 and the effector-caspase 3. As shown in Fig. 1A, no caspase activity over background was detectable in TNF/CHX/zVAD-treated cells, whereas the proapoptotic stimulus TNF/CHX as a control strongly activated both caspases. To monitor ceramide-mediated ciPCD, we decided to analyze distal events in the pathway (changes in cell morphology, loss of membrane integrity), since it was not clear whether Bcl-2 would affect proximal steps of the signaling chain (e. g. the generation of the lipid ceramide [31]). When analyzed microscopically, both untreated cell lines uniformly displayed an intact cell morphology with round cells and almost no irregular shapes or cell debris (Fig. 1B, left panels). As expected, induction of ciPCD by TNF/CHX/zVAD strongly increased the amount of Jurkat wildtype cells with clear morphological irregularities and the "fried egg-like" shape characteristic for necrosis-like ciPCD (Fig. 1B, upper right panel; [32]). In contrast, the majority of wildtype-Bcl-2-overexpressing Jurkat cells retained an intact cell morphology despite treatment (Fig. 1B, lower right panel). As a more objective parameter for ciPCD, we measured uptake of PI as a marker for loss of plasma membrane integrity in dying cells. Again, wildtype-Bcl-2-overexpressing Jurkat cells clearly displayed a higher resistance to TNF/CHX/zVAD-induced ceramide-mediated ciPCD than their parental counterparts (Fig. 1C, D), demonstrating that in addition to its anti-apoptotic properties, wildtype Bcl-2 also can protect against ciPCD. Our results are supported by studies from other groups demonstrating that overexpression of wildtype Bcl-2 can delay the onset of ceramide-induced ciPCD in yet other cell types, such as L929 fibrosarcoma cells [33]. As a possible mechanism, Denecker and coworkers have suggested a prolonged integrity of mitochondrial oxidative phosphorylation or complexation of the proapoptotic protein BNIP3 by wildtype Bcl-2 [34]. Moreover, our results implicate that both mitochondria and the ER represent target organelles of wildtype Bcl-2 that may participate in ceramide-mediated ciPCD.

### Transient expression of Bcl-2 constructs with restricted subcellular localization

**Figure 2:**
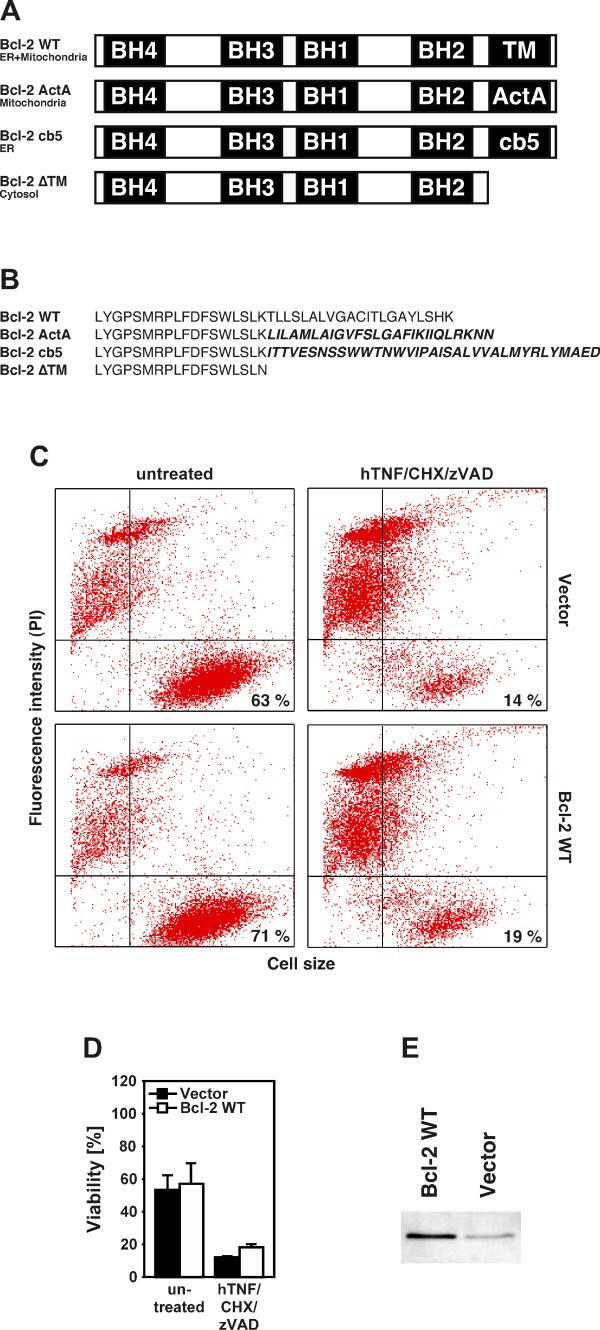
**Transient expression of Bcl-2 constructs with defined subcellular localization in Jurkat cells**. (A) Schematic representation of human wildtype Bcl-2 (Bcl-2 WT) localizing to both mitochondria and the ER, Bcl-2 ActA targeted to mitochondria, Bcl-2 cb5 expressed at the ER, and Bcl-2 ΔTM localized in the cytosol. The Bcl-2 homology domains BH1-BH4 are indicated together with the transmembrane domain (TM) for wildtype Bcl-2, which is replaced by amino acids from *Listeria monocytogenes *ActA, from rat cytochrome b5 (cb5) or deleted altogether in the other constructs. (B) amino acid sequence of the carboxyterminus of wildtype Bcl-2, Bcl-2 ActA, Bcl-2 cb5 and Bcl-2 ΔTM. The amino acids derived from ActA and cb5 are shown in bold. (C) Wildtype Jurkat cells were transiently nucleofected with empty vector pRc/CMV or with pRc/CMV encoding wildtype Bcl-2. 24 h after transfection, the cells were stimulated with 100 ng/ml hTNF in combination with 5 μg/ml CHX and 50 μM zVAD-fmk or left untreated for another 24 h. Prior to stimulation, the cells were preincubated for 60 min with 50 μM zVAD-fmk. PI-uptake was determined by flow cytometry and the percentage of viable cells is indicated in the lower right quadrants of the dot plots. One representative experiment out of three performed is shown. (D) Quantification of cell viability data. The bar graphs represent the means from all three independent experiments, error bars indicate the respective standard deviations. Due to the transfection procedure, the cells generally display a lower viability than untransfected cells (Fig. 1). (E) In parallel, expression of Bcl-2 in the transfectants was visualized by Western blot analysis. The band in vector transfectants represents endogenous Bcl-2.

Since the above results did not yet provide information on a specific contribution of each organelle, we made use of a panel of Bcl-2 constructs that are specifically expressed in distinct cellular compartments. The carboxyterminus of wildtype Bcl-2 contains a stretch of hydrophobic amino acids (the "insertion sequence") that has been proposed to anchor proteins in the cell membrane. Previously, Zhu and coworkers have replaced this natural 21 amino acid insertion sequence of wildtype Bcl-2 by the analogous 26 amino acid insertion sequence from *Listeria monocytogenes *ActA to target Bcl-2 specifically to the outer mitochondrial membrane (Bcl-2 ActA, Fig. 2A, B). Similarly, the construct Bcl-2 cb5 (Fig. 2A, B) contains the 35 amino acid insertion sequence of the ER-specific isoform of rat hepatic cytochrome b5, directing Bcl-2 to the cytosolic face of the ER. In addition, Zhu and coworkers constructed a gene encoding a form of Bcl-2 that lacks the hydrophobic membrane-anchoring sequence (Bcl-2 ΔTM, Fig. 2A, B) and which localizes to the cytosol [16]. We transiently nucleofected wildtype Jurkat cells with the vector pRc/CMV encoding wildtype Bcl-2 or with empty pRc/CMV and determined their resistance against ceramide-induced ciPCD. In analyses for PI-uptake, cells transfected with wildtype Bcl-2 generally displayed a slightly higher viability than vector-transfected cells (this was seen for untreated as well as TNF/CHX/zVAD-treated cells). However, this difference was only marginal, and no pronounced protection from ciPCD was seen in comparison to vector-transfected control cells (Fig. 2C, D). These results were confirmed in independent experiments utilizing cotransfection of green fluorescent protein as a marker for transfection efficiency (data not shown). Since we have previously found that transient transfection of Jurkat cells often occurs with low efficiency, we generated lysates from the transfectants and analyzed them by Western blot. As shown in Fig. 2E, Jurkat cells transfected with wildtype Bcl-2 showed only a limited overexpression of the construct relative to the endogenous Bcl-2 protein present in vector-transfected control cells, thereby explaining their inadequate protection.

### Wildtype, but not organelle-specific Bcl-2 protects from ceramide-mediated ciPCD in stably transfected Jurkat cells

**Figure 3:**
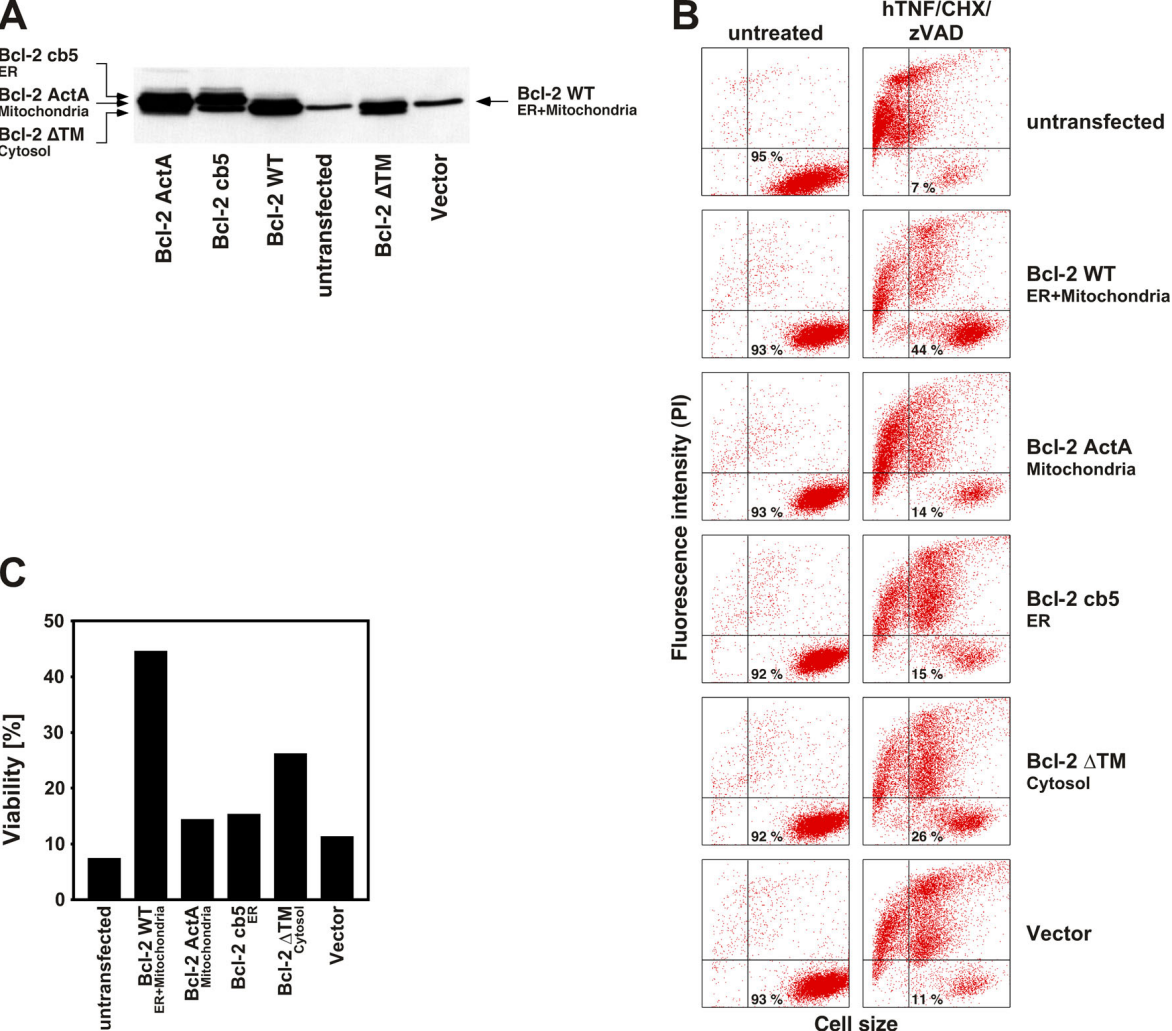
**Wildtype, but not organelle-restricted Bcl-2 protects stably transfected Jurkat cells from ceramide-mediated ciPCD**. (A) Expression of pRc/CMV-encoded Bcl-2 WT, Bcl-2 cb5, Bcl-2 ActA and Bcl-2 ΔTM-constructs in stably transfected Jurkat cells. Cell lysates were prepared from untransfected Jurkat cells or Jurkat cells stably transfected with empty vector or with Bcl-2 constructs targeted to the ER, mitochondria, both, or the cytosol as indicated. Expression of the constructs was verified by immunoblot with a Bcl-2-specific antibody (sc-7382, Santa Cruz). Multiple bands result from detection of the endogenous Bcl-2 protein in addition to the construct (see untransfected Jurkat cells). (B) Flow cytometric analysis of PI-uptake in untransfected and stably transfected Jurkat cells. Prior to stimulation, the cells were preincubated for 60 min with 50 μM zVAD-fmk. After that, ceramide-mediated ciPCD was induced by treatment with 100 ng/ml hTNF in combination with 5 μg/ml CHX and 50 μM zVAD-fmk for 48 h, or the cells were left completely untreated. The percentage of viable cells is shown in the lower right quadrants of the dot plots. (C) Bar graphs showing the fraction of viable cells for each of the stably transfected lines depicted in (B). Similar results were obtained in two additional experiments with different incubation times (24 and 72 h), although with increased or reduced overall viability (data not shown).

We therefore generated Jurkat cells that stably overexpress the above panel of organelle-specific pRc/CMV-Bcl-2 constructs [21]. For each transfectant cell line, we confirmed overexpression of the corresponding Bcl-2 construct by Western blot analyses (Fig. 3A). We additionally verified the assumed subcellular localization of Bcl-2 for each stably transfected cell line by confocal laser scanning microcopy as described elsewhere [21]. In these experiments, only the ER-specific mutant Bcl-2 cb5, but not mitochondria-targeted Bcl-2 ActA or cytosol-directed Bcl-2 ΔTM colocalized with the endoplasmic calcium ATPase SERCA. Likewise, only Bcl-2 ActA, but not Bcl-2 cb5 or Bcl-2 ΔTM colocalized with mitochondrial cytochrome c, whereas Bcl-2 ΔTM displayed the expected diffuse expression pattern in the cytosol, but also localized to the nucleus [21]. Having verified the integrity of the generated stable transfectants, we monitored their resistance against ceramide-induced ciPCD. As shown in Fig. 3B and Fig. 3C, all untreated samples uniformly displayed a high percentage of viable cells after 48 h (Fig. 3B, left panels). Induction of ciPCD by TNF/CHX/zVAD, however, resulted in an almost complete loss of viability in untransfected cells. Jurkat cells stably overexpressing pRc/CMV-encoded wildtype Bcl-2 were clearly protected against ciPCD, identical to Jurkat cells stably overexpressing wildtype Bcl-2 from the distinct construct pSFFV- Bcl-2 (Fig. 1). Also, Jurkat cells expressing only the vector control pRc/CMV did not significantly differ in their response from untransfected Jurkat cells, confirming that the observed resistance of Bcl-2 WT-overexpressing cells was genuine and not due to artifacts caused by the transfection/stable selection procedure. Remarkably, the transfectants overexpressing mitochondrially and ER-targeted Bcl-2 (ActA and cb5) were not substantially better protected than the vector controls. Likewise, overexpression of cytosolic Bcl-2 ΔTM resulted only in slightly better protection from ceramide-elicited ciPCD, however not nearly at the level of wildtype Bcl-2 (Fig. 3B, C). Similar results were obtained at 24 and 72 h of incubation, although with increased or reduced overall viability, and with Bcl-2 ΔTM showing a protection even more comparable to Bcl-2 ActA and cb5 (data not shown), in summary arguing that efficient protection from ceramide-mediated ciPCD is conferred only by wildtype Bcl-2, but not by organelle-restricted expression of Bcl-2.

## Discussion

In this study, we show for the first time that Bcl-2-mediated protection from non-apoptotic, caspase-independent PCD requires the full-length, wildtype protein whereas a limited expression of Bcl-2 at mitochondria, the ER, or the cytosol/nucleus is not sufficient to prevent ciPCD elicited by ceramide. The failure of the organelle-specific constructs to confer protection is not due to an inefficient level of expression (Fig. 3A) or an incorrect localization [21], and their functionality has been previously demonstrated for apoptotic PCD using the same stable transfectants as analyzed here [21,22]. Moreover, the protection by wildtype Bcl-2 was independently seen in two distinct stably transfected Jurkat lines, expressed from different vector backbones (pSFFV-neo, pRc/CMV).

The fact that expression of Bcl-2 restricted to mitochondria, the ER (or the cytosol/nucleus, if the results with Bcl-2 ΔTM are additionally taken into account) is not effectively suppressing ceramide-induced ciPCD may suggest that wildtype Bcl-2 interferes with this form of PCD at yet another cellular site. However, this is unlikely, as wildtype Bcl-2 has been detected only at mitochondria, the ER and the nucleus in numerous previous studies (reviewed in [15,35]). Alternatively, the exclusive protection by wildtype Bcl-2 may be explained by a combined requirement of Bcl-2 at both mitochondria and at the ER, suggesting that both organelles coordinately participate in ciPCD via a molecular crosstalk. This hypothesis appears especially attractive because a similar crosstalk has been shown for apoptotic PCD, where the ER controls mitochondrial apoptosis by releasing proapoptotic calcium [15]. Although calcium likewise appears as an attractive candidate molecule for ER-mitochondrial crosstalk in ciPCD (especially when considering that the calcium-dependent calpain proteases can elicit ciPCD [2]), the precise nature of this crosstalk will have to be clarified in future studies.

Notably, in autophagy, a distinct form of ciPCD, Bcl-2 has been recognized to act as a negative regulator specifically at the ER by binding to the autophagy-inducing protein Beclin-1 [15].

Even though the role of Bcl-2 has been most intensively studied in mitochondria and the ER, Bcl-2 also localizes to the nucleus. However, apart from a recent study where it was shown that nuclear Bcl-2 forms an integral part of mitotic chromosomes [36], information on the functions of nuclear Bcl-2 are scarce. Therefore, nuclear functions of Bcl-2 may likewise be required for suppression of ciPCD in concert with its mitochondrial and ER-specific functions.

In summary, the further exploration of the exact roles of mitochondrial, ER- and nucleus-specific Bcl-2 as well as their potential crosstalk will provide further insight into the molecular mechanisms by which ceramide-mediated ciPCD is executed. At the clinical level, this may directly prove beneficial also for radiation therapy by providing novel options to eliminate tumor cells that have become resistant to apoptotic death signals.

## Conclusion

**Figure 4:**
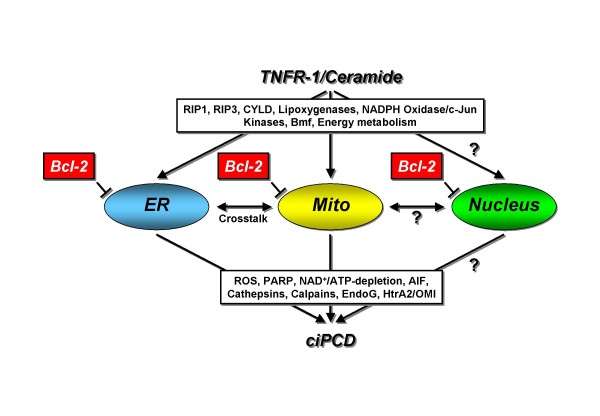
**Overview scheme depicting the proposed requirement of the ER and mitochondria in TNF-R1/ceramide-mediated ciPCD**. Known proximal mediators of TNF-R1/ceramide-induced ciPCD are indicated, as are mediators of ciPCD that potentially act downstream of the ER and mitochondria. Only wildtype Bcl-2 simultaneously acting at the ER, at mitochondria and at the nucleus efficiently blocks the caspase-independent death signals of TNF-R1/ceramide, whereas Bcl-2 constructs specifically localizing to each organelle do not prevent ciPCD. This suggests that the corresponding signaling pathways of TNF-R1/ceramide target both the ER and mitochondria, and that both organelles participate in ciPCD via a molecular crosstalk. The nucleus may represent a further organelle that participates in these signaling pathways, yet its role remains to be confirmed.

Based upon the data presented, we conclude that expression of Bcl-2 at both the ER and mitochondria (and possibly the nucleus) is required for effective suppression of ceramide-mediated caspase-independent programmed cell death. This also implicates the participation of both (or all three) organelles in the corresponding signaling pathways and suggests a molecular crosstalk between the ER, mitochondria (and the nucleus). A corresponding overview scheme is given in Fig. 4.

## Competing interests

The authors declare that they have no competing interests.

## Authors' contributions

AD carried out immunoblots, transient transfections, flow cytometric analyses and analyzed data. JS carried out additional flow cytometry, morphological analysis by microscopy and analyzed data. LT analyzed caspase activity. CB provided critical reagents and participated in the design of the study. DA conceived and designed the experiments, analyzed data and wrote the paper. All authors read and approved the final manuscript.
